# Wearable Device Heart Rate and Activity Data in an Unsupervised Approach to Personalized Sleep Monitoring: Algorithm Validation

**DOI:** 10.2196/18370

**Published:** 2020-08-05

**Authors:** Jiaxing Liu, Yang Zhao, Boya Lai, Hailiang Wang, Kwok Leung Tsui

**Affiliations:** 1 School of Data Science City University of Hong Kong Kowloon China (Hong Kong); 2 Centre for Systems Informatics Engineering City University of Hong Kong Kowloon China (Hong Kong)

**Keywords:** sleep/wake identification, hidden Markov model, personalized health, unsupervised learning, sleep, physical activity, wearables, heart rate

## Abstract

**Background:**

The proliferation of wearable devices that collect activity and heart rate data has facilitated new ways to measure sleeping and waking durations unobtrusively and longitudinally. Most existing sleep/wake identification algorithms are based on activity only and are trained on expensive and laboriously annotated polysomnography (PSG). Heart rate can also be reflective of sleep/wake transitions, which has motivated its investigation herein in an unsupervised algorithm. Moreover, it is necessary to develop a personalized approach to deal with interindividual variance in sleep/wake patterns.

**Objective:**

We aimed to develop an unsupervised personalized sleep/wake identification algorithm using multifaceted data to explore the benefits of incorporating both heart rate and activity level in these types of algorithms and to compare this approach’s output with that of an existing commercial wearable device’s algorithms.

**Methods:**

In this study, a total of 14 community-dwelling older adults wore wearable devices (Fitbit Alta; Fitbit Inc) 24 hours a day and 7 days a week over period of 3 months during which their heart rate and activity data were collected. After preprocessing the data, a model was developed to distinguish sleep/wake states based on each individual’s data. We proposed the use of hidden Markov models and compared different modeling schemes. With the best model selected, sleep/wake patterns were characterized by estimated parameters in hidden Markov models, and sleep/wake states were identified.

**Results:**

When applying our proposed algorithm on a daily basis, we found there were significant differences in estimated parameters between weekday models and weekend models for some participants.

**Conclusions:**

Our unsupervised approach can be effectively implemented based on an individual’s multifaceted sleep-related data from a commercial wearable device. A personalized model is shown to be necessary given the interindividual variability in estimated parameters.

## Introduction

### Background

Sleep plays a vital role in maintaining health [1,2]. Adequate sleep can help to maintain a high quality of life [3]. In contrast, short sleep duration may lead to adverse health outcomes, such as obesity, insulin resistance, depression, hypertension, and cardiovascular disease [4-8]. Changes in sleep duration have been associated with declined cognitive function and increased mortality rate in middle-aged population [9,10]. Thus, to detect changes in sleep patterns early on or for sleep disorder diagnosis, it is essential to measure sleep duration accurately and longitudinally.

Polysomnography is the gold standard for sleep duration and sleep quality assessment; various devices are used to record multiple body functions such as brain activity, eye movements, skeletal muscle movement, and heart rhythm during sleep, and it is typically done in a sleep lab or clinic [11]. Health care professionals use these physiological measures to assess sleep states, however, the cost of overnight PSG may range from US $600 to $5000 each night [12]. Such assessment is expensive and intrusive for consumers, and labor-intensive and resource-demanding for health care professionals to achieve [13-15], making it hard to use for long-term sleep monitoring at home.

An emerging trend has been to adopt sensor-based wearable devices to assess sleep duration and achieve long-term sleep monitoring. According to the reports from the International Data Corporation, 83.8 million wearable devices were shipped during the first two quarters of 2019 [16,17]. One of the most common technologies for consumer sleep-monitoring wearable devices is accelerometer-based actigraphy [18], which tracks physical movement and determines when a person is asleep or awake based on whether a low or high amount of activity is recorded [19,20].

### Related Work

A recent paper [21] reviewed and validated existing supervised sleep-scoring algorithms using actigraphy in a large cohort. Many types of supervised algorithms have been used, such as linear discriminative analysis [22,23], decision trees [24], artificial neural networks [24], long short*-*term memory [21], and convolutional neural networks [21]; however, all require training with annotated PSG, collected and labeled at great expense. Moreover, the labeled daily-living sleep/wake PSG data are challenging to collect during daytime making 24-hour accuracy hard to evaluate.

Because annotated PSG is hard to acquire for model building, it is intuitive to use an unsupervised method. Commonly used techniques are based on rules and thresholds. For instance, the Actiwatch (Mini Mitter Co Inc) software determined the start of sleep when there were 10 consecutive minutes below a certain mobility threshold and determined the end of sleep with 10 consecutive minutes above the threshold [25]. This approach was commonly adopted in commercial wearable devices because of its simplicity; however, the choice of the mobility threshold was relatively arbitrary. Few studies have used other unsupervised machine learning approaches such as clustering [15] and hidden Markov model (HMM) [26].

Hidden Markov models are well suited to modeling time series data in a probabilistic way by using latent states [27]. Temporal dependency can be learned, and the parameters of the model are interpretable. In the context of bioinformatics, HMMs have been applied to monitor circadian rhythmicity using physical activity data to characterize interindividual variability [28]. In other high-frequency physiological data collected during PSG—such as electroencephalography, electrooculography, and electromyography—HMMs have been used to classify sleep stages [29,30]. These papers showed that the sleep/wake cycle could be inferred from physiological data using HMMs; therefore, we wanted to extend this approach and its strengths to activity and heart rate data from wearable devices.

In addition to modeling sleep/wake transitions via activity data, heart rate is also reflective of the circadian cycle, and it can be recorded accurately using photoplethysmography in wearable devices [31]. In a 24-hour assessment study in ambulatory patients, heart rate varied significantly in sleep and wake periods [32]. Willemen et al [33] showed that heart rate, along with activity, can predict sleep and wake well with the use of support vector machine algorithms on healthy participant data; however, this approach also required supervised training (ie, labeled PSG).

Furthermore, the generalizability of existing algorithms is of concern especially when different sleeping patterns and habits can be observed in different people [15]. Person-to-person differences in demography and physiology can have significant effects on sleep/wake patterns and characteristics [34,35]. Personalized sleep-scoring algorithms are needed to avoid interindividual variance problems, since algorithms learn from individual lifestyle and physiological patterns using long-term contextual history. Existing studies have shown that the sleep/wake state can be better inferred using a personalized approach from actigraphy [36,37].

### Objectives

In this paper, we aimed to explore the feasibility of using HMM to analyze heart rate and activity data collected by a wearable device and to develop a personalized and unsupervised sleep/wake identification approach. To our knowledge, there is little research focused on personalized and unsupervised sleep/wake identification algorithms using a wearable device. Also, the approach enables advantageous complementary use of both heart rate and activity data. The algorithm is demonstrated using a real-world data set collected with commercial wearable devices in the older adult population and its performance is illustrated with case studies.

We describe recruitment and data collection, data preprocessing steps, HMM, rescoring rules, and comparison scoring results. We also demonstrate the approach with a case study, investigate the fusion effect of heart rate and activity data in modeling, compare our scoring results with Fitbit’s results individually, and investigate pattern changes using daily models.

## Methods

### Participant Recruitment and Data Collection

We collaborated with an older adult care center in Hong Kong to recruit participants who met the following criteria: aged 60 years or above, community-dwelling Hong Kong residents, and willing to take participate in a 3-month longitudinal observational study from December 18, 2017 to February 28, 2018. The Research Ethics Committee of the City University of Hong Kong approved this pilot study (reference number 3-2-201803_02). All participants provided written consent.

Heart rate data and activity data were collected using Fitbit Alta (Fitbit Inc). Participants were asked to wear the device on their nondominant hand for the full 24 hours each day for 3 consecutive months. For activity data, the most common choice for use in sleep/wake classification algorithms is activity count. However, since activity count was unavailable in Fitbit, we used step count instead, which was also a reflection of activity intensity in an epoch. Fitbit Alta reported heart rate every 1 minute and reported step count every 15 minutes.

### Data Preprocessing

Since the study was conducted in a free-living home environment, some participants removed the devices when showering or at night. When the participant removed the device, the device reported heart rate and step count as zero, and the nonwear time could be inferred. The recordings were examined, and nonwear days were identified and removed before analysis if (1) more than 30 minutes of heart rate data were in that day or (2) there was a step count of zero on that day. We excluded participants who had more than 50% of the days in the observation period identified as nonwear from analysis.

After the elimination of nonwear days, any remaining data with missing values could be kept and dealt with in HMM. Next, we further preprocessed the step count data. In order to facilitate the fusion of step count and heart rate data in the models, downscaling was used to deal with the multigranularity data [38]. It was achieved by disaggregating the 15-minute step count data and simulating the of 1-minute step count time series. We assumed that the 15-minute step count U^STEP^ was evenly distributed to every minute. Thus, 1-minute step counts were generated by


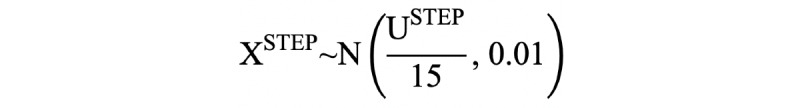
 (**1**)

The total step count in 15 minutes was closely preserved.

### Hidden Markov Models

#### Definitions

**Figure 1:**
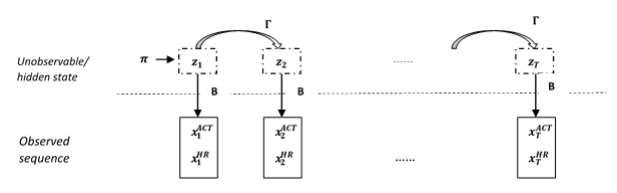
An illustration graph of the structure of multivariate hidden Markov model.

Hidden Markov models are composed of paired stochastic variables: hidden states and observed variables. The model assumes that an observed sequence has been generated by distributions, which conditionally depend on the hidden states in an underlying and unobserved Markov process. In our sleep/wake identification problem, we considered two-state hidden Markov models. The hidden states were S={s_1_, s_2_}. Each observed bivariate time series (of heart rate and activity data) was denoted as


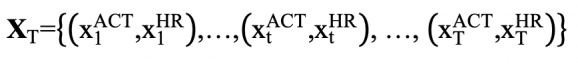
 (**2**)

where t ∈ {1,...,T} and T was the total length. The two-state chain was initialized by the initial state distribution, π={ π_1_, π_2_} where Σπ_k_=1. The sequence of hidden states was **Z_T_**={z_1_,z_2_,...,z_T_}, where z_t_ ∈ S for any t. The structure of a standard multivariate HMM is shown in Figure 1.

The unobserved process was assumed to satisfy the Markov property. The transition probability matrix was denoted by **Γ** as


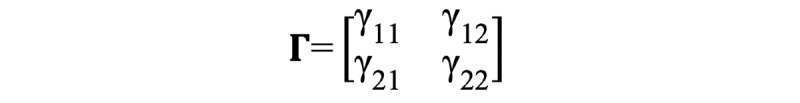
 (**3**)

whose (i,j) entry represented the probability of state s_i_ transitioning to state s_j_:


P(z_t+1_=s_j_ | z_t_=s_i_) = γ_ij_, s_i,_ s_j_ ∈ S (**4**)

The emission density function


p(x_t_ | Z_t_=s_i_) (**5**)

was associated with hidden states, which denoted the density of the observation **X**_T_ if the hidden state was s_i_ at time t. For multivariate time series, there were 2 schemes to develop HMMs: (1) Specify the state-dependent joint distributions of the observed variables for different states or (2) assume contemporaneous conditional independence.

#### Model Scheme M1: Specification of the State-Dependent Joint Distributions

In the multivariate case, it can be straightforward to specify the joint distribution in the context of our application, since heart rate is highly correlated with activity intensity. The bivariate normal distribution was considered because of its practical uniqueness. Thus, the emission density function could be written as follows if we assumed a bivariate joint distribution for **x**_t_ | s_i_:


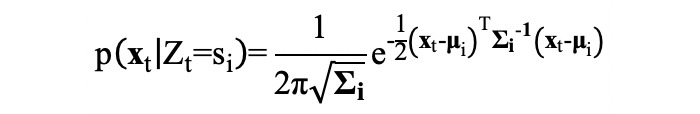
 (**6**)

with


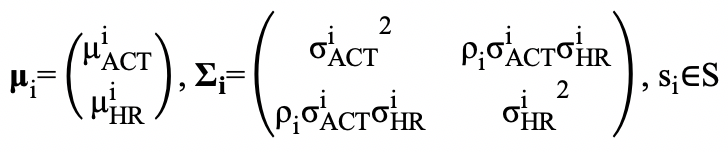
(**7**)

The correlation between heart rate and activity level can be directly characterized by *ρ*_i_.

#### Model Scheme M2: Contemporaneous Conditional Independence

Specifying suitable joint distributions can be sometimes difficult, and for simplicity, contemporaneous conditional independence can be assumed. This means that the state-dependent joint distribution is the product of the corresponding marginal distributions:


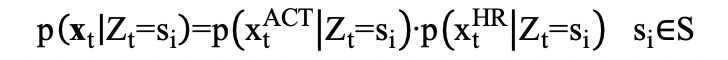
 (**8**)

Note, contemporaneous conditional independence does not mean the two observed time series are mutually independent since the Markov chain can induce dependent pairs [27], and the marginal distributions need not necessarily belong to the same family of distribution. Thus, we can assume the univariate distributions in different states according to prior information. The choices of distributions are discussed in our real-world case study.

### Model Fitting and Decoding

After the model was fully specified. The likelihood could be obtained by summing the values assumed by z_1_,z_2_,...,z_T_:


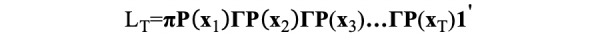
(**9**)

The likelihood function was evaluated by the forward algorithm. An advantage of the HMM is that missing data can be dealt with simply by adjusting the likelihood computation. The corresponding state-dependent probabilities were replaced by 1 for all states. Parameter estimation was achieved by numerical maximization or Baum-Welch algorithm [27]. Next, we decoded the series of the hidden states globally by maximizing the conditional probability of the whole sequence P(**Z**_T_ | **X**_T_). The optimal path was found using the Viterbi algorithm with estimated parameters. The hidden states were matched with sleep and wake states. The state with higher estimated mean heart rate, *µ*_HR_, and mean activity level, *µ*_ACT_, represented the active status of the participants, and related to a waking state. On the other hand, the state with lower estimated mean heart rate and mean activity level represented the resting status, which was related to a sleeping state (heart rate variance, *σ*^2^_HR_, and activity level variance, *σ*^2^_ACT_).

### Implementation of the Hidden Markov Models

**Figure 2:**
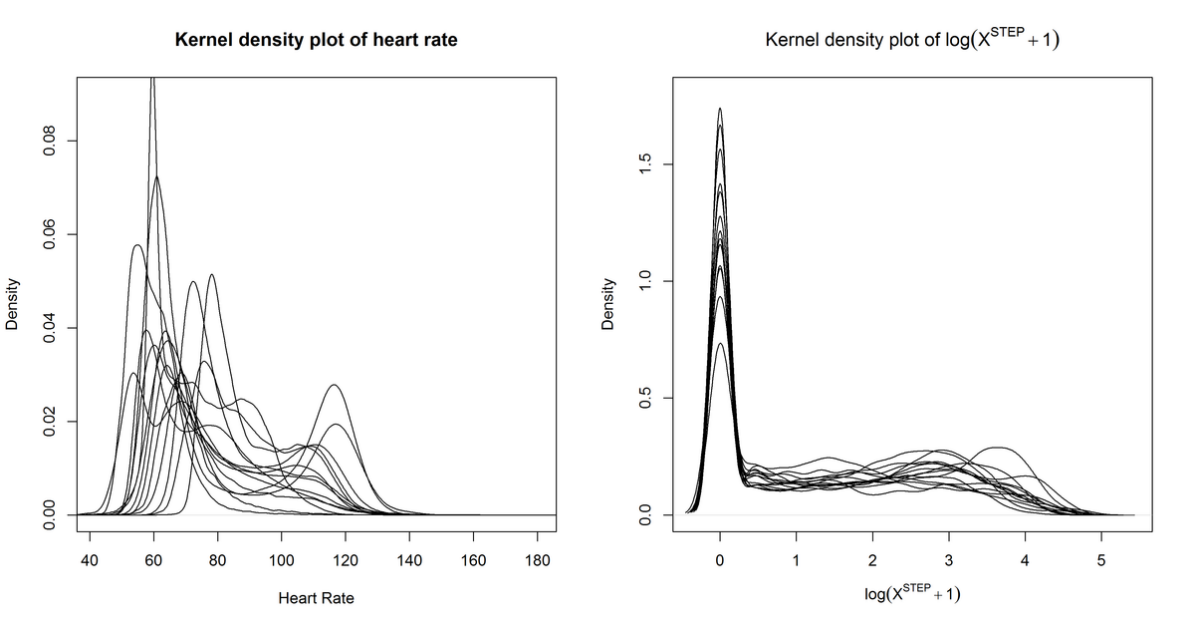
Kernel density plots for heart rate and log(X_STEP+1) for all participants.

After preprocessing, the data were ready for implementation of the HMMs. For each participant, we plotted the kernel density estimates of heart rate and of log-transformed step count (Figure 2) to explore suitable prior joint emission distributions. From the kernel density plot of heart rate, the overdispersed and nonsymmetric observations suggested a bimodal distribution, which may be modeled by a mixture model, and very likely a two-component Gaussian mixture; however, mixture models do not take temporal dependency into account, which prompted us to adopt the hidden Markov model. A Poisson distribution is a natural choice for modeling count data; however, the step count ranged from 0 to 160. Since it would have been computationally expensive to estimate, especially for our long sequence, we took the log transformation of step count and found that they were also distributed marginally as a two-component Gaussian mixture.

Based on the marginal density plots, we proposed two schemes for fitting multivariate HMM. For model scheme M1, we specified bivariate Gaussian distribution for heart rate and log(X^STEP^+1) for both states; for model scheme M2, we assumed univariate Gaussian distribution for heart rate for both states and univariate Gaussian distribution for log-transformed step count for both states.

Two HMMs were fitted for each participant, one HMM modeled using model scheme M1 and another using model scheme M2. The best model was chosen based on Akaike information criterion (AIC) and Bayesian information criterion (BIC). Moreover, the goodness of fit of the best model was further assessed by ordinary normal pseudo-residuals [27]:


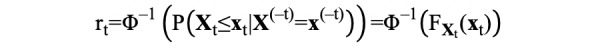
 (**10**)

If the observations **x**_1_,...,**x**_T_ were indeed generated by the model **X**_t_∼**F**_t_, the ordinary normal pseudo-residuals would be distributed standard normal.

With the estimated parameters for the best model, we found a globally optimal path for the observations. The decoding of the hidden states was passed to rescoring rules, and the final scoring results of sleep and wake states were decided. The implementation of HMM was built based on the dependent mixture models package (depmixS4; version 1.4-2) in R software (version 3.5.1) [39]

To study the fusion effect of heart rate and activity in HMM, two HMM models observing single source data was considered for comparison. We fitted a two-state hidden Markov model observing activity level X^ACT^, which was log(X^STEP^+1), denoted as the activity HMM, and a model observing heart rate denoted as the heart rate HMM. In both the activity HMM and the heart rate HMM, the emission distributions for both states were assumed to be normal distributions according to the empirical data analysis.

### Rescoring Rules

In actigraphy algorithms, Webster et al [40] reported that the most common error was scoring wake as sleep. In order to correct this systematic error, they developed these rescoring rules, which were further validated by different researchers [21,22]. Webster rescoring rules can be described as (1) after at least 4 minutes scored as wake, the first minute scored sleep will be rescored wake; (2) after at least 10 minutes scored as wake, the first 3 minutes scored sleep will be rescored wake; (3) after at least 15 minutes scored as wake, the first 4 minutes scored sleep will be rescored as wake; (4) 6 minutes or less scored sleep surrounded by at least 10 minutes (before or after) scored as wake are rescored wake; and (5) 10 minutes or less scored as sleep surrounded by at least 20 minutes (before or after) scored as wake are rescored wake. These rules were applied to 1-minute decoding results from HMM sequentially.

**Figure 3:**
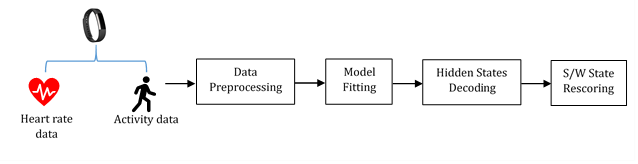
The workflow of our hidden Markov model–based sleep/wake identification approach. S/W: sleep/wake.

The workflow of our sleep/wake identification approach can be summarized in Figure 3.

### Comparison of Scoring Algorithms

Fitbit Alta automatically detects sleep based on activity—“When your body is completely at rest and you haven’t moved for about an hour, your Fitbit device records that you’re asleep” [41];—however, the exact scoring algorithm is proprietary. Only the sleep/wake scoring results can be compared with Fitbit’s output (7 sleep-related states). For comparison, *asleep*, *deep*, *light*, and *REM* were reclassified as *sleep*, while *restless*, *awake*, and *wake* were reclassified as *wake*.

### Daily Basis Model

In addition to applying the proposed model to 3-month time periods for each participant, as a pilot experiment, we applied the personalized algorithms on a daily basis for each person. To investigate whether there was any difference in estimated parameters between weekdays (Monday to Friday) and weekends (Saturday and Sunday), we used two-tailed independent *t* tests to compare the parameters of the two for each participant (*P*<.05 was deemed significant).

## Results

### Overview

**Figure 4:**
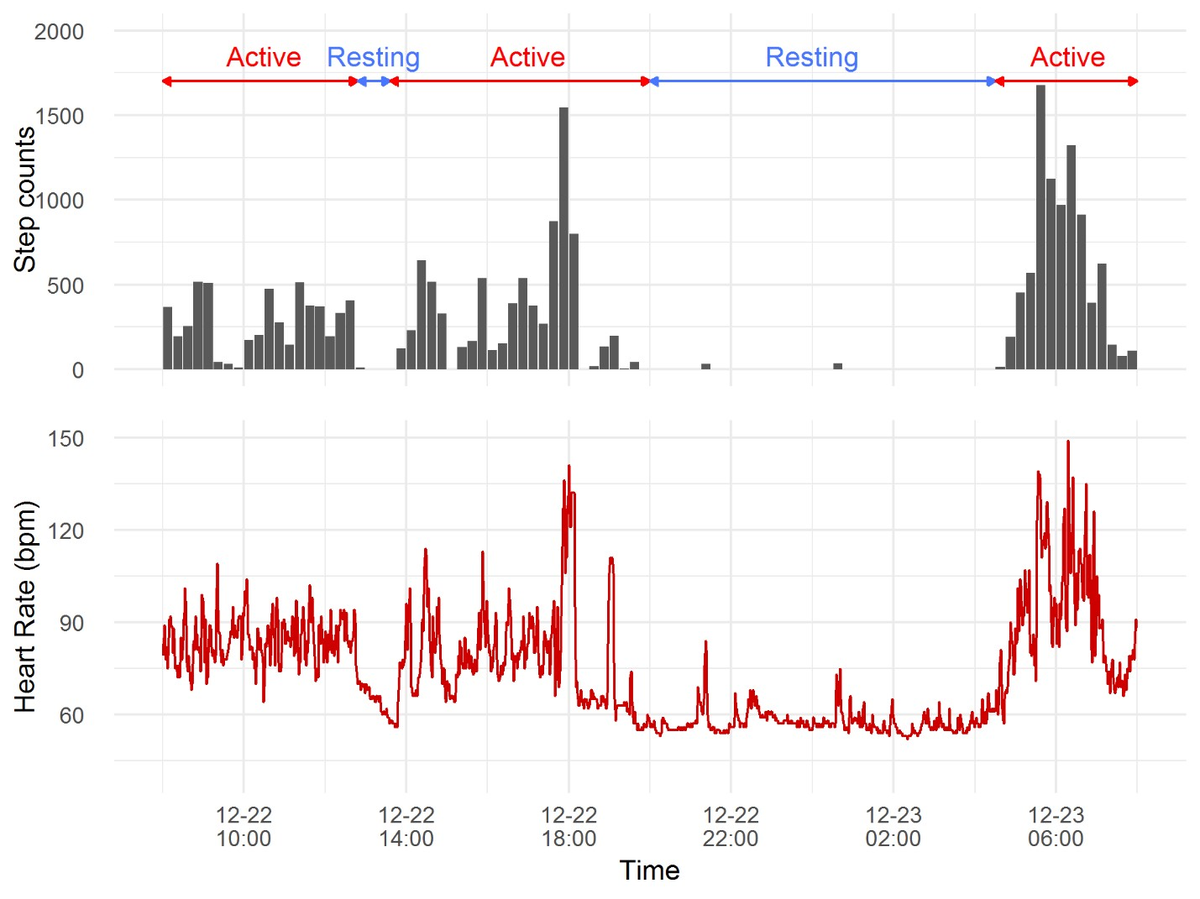
A 24-hour example plot of step count every 15 minutes and heart rate every 1 minute for participant EL01 from 8 AM to 7:59 AM the following morning. Note: Times are in 24-hour format.

After nonwear exclusion, there were 14 participants whose data qualified for analysis (aged from 61 to 91 years old; 12 women and 2 men); 6 participants had hypertension, 5 had high cholesterol, 2 had diabetes mellitus, 3 had cancer, and 1 had a stroke. The percentage of missing heart rate data ranged from 0.31% to 0.96% (mean 0.64%). Examples of 24 hours of step count and heart rate data are shown in Figure 4 in which the circadian cycle was quite clearly evident, and the segmentation of activity and heart rate over the 24 hours was visible.

### Model Selection and Parameter Estimation Results

In order to illustrate our proposed approach, we used the sleep and wake identification of two typical examples, participants EL02 and EL21, for demonstration. We first examined the recordings to eliminate nonwear days. There were 16 days were eliminated from analysis for EL02 and 9 days were eliminated from analysis for EL21.

To compare two schemes of modeling multivariate time series, we present model selection results, including log likelihoods, AIC, and BIC in Table 1. According to both AIC and BIC, the model with bivariate normal joint distribution specified for both states was more appropriate for EL02. For the other 13 participants, both AIC and BIC also tended to favor model scheme M1, which assumed bivariate normal emission distributions for both states.

**Table 1:** Comparison of models fitted to heart rate and log(XSTEP+1) for EL02.

Model scheme	Emission distribution	*df*	Log likelihood	AIC	BIC
M1	Bivariate normal	13	–419428.3	838882.7	839004.3
M2	Conditional independence	11	–424798.5	849721.9	849721.9

**Figure 5:**
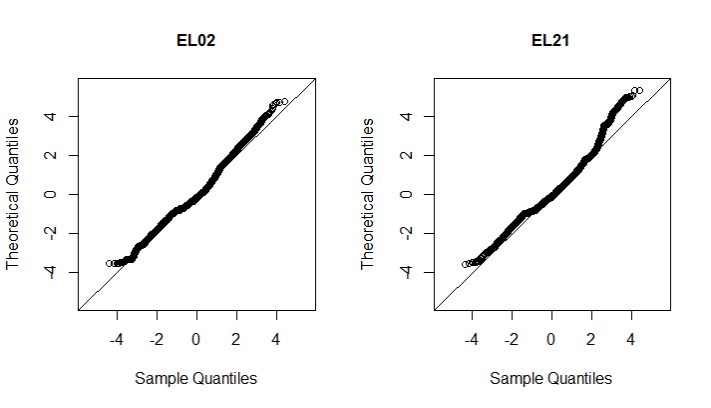
Quantile-quantile plots of ordinary normal pseudo-residuals in model scheme M2 for EL02 and EL21.

We checked the general goodness of fit of the best model with ordinary normal pseudo-residuals. If the fitted model was valid, the pseudo-residuals would be distributed normally. By visual inspection of the quantile-quantile (QQ) plot of the pseudo-residuals for each participant’s best model, we found they fit well. We present QQ-plots for EL02 and EL21 in Figure 5; we could observe that for EL02 and EL21 the pseudo-residuals were, in general, distributed normally, though the distribution had heavier tails for EL21. Overall, our 2-state HMM model assuming bivariate Gaussian was adequate and valid for the participants in our study.

The estimated parameters in emission distributions for EL02 are shown in Table 2 for the best model chosen by AIC and BIC. According to the estimated parameters for emission distribution in different states, we can generally classify the 2 hidden states as sleep and wake. Wake was the state with higher mean heart rate and mean activity level in the emission density distribution. In the wake state, the estimated variance for heart rate was 213.68 and for activity level was 0.83, which were much larger than those of 157.37 for heart rate and 0.15 for activity level during the sleep state. This reflected the variability of activities during the participant’s waking period. The estimated transition probability for EL02 was as follows:


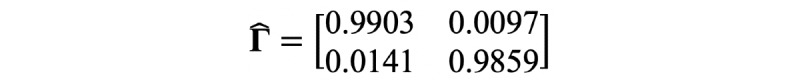


From the estimated transition probability, it was more likely that, in a given minute, the participant would stay in the same state as the state in the previous minute. It also supported the necessity of the use of HMM to deal with the time dependence in observations.

For participant EL21, the estimated parameters in emission density distributions are also shown in Table 2. The transition probability for EL21 was estimated as


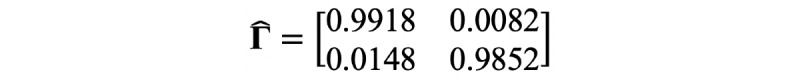


The general pattern of estimated parameters for EL21 was similar to EL02. While the difference between estimated mean values in the two states were much lower than that of EL02. For the sleep state, the activity level was roughly zero (*µ*_ACT_ <0.01). For the wake state, the heart rate (*µ*_HR_=80.17) and the activity level (*µ*_ACT_=2.22) were lower than those for EL02, showing that the mean intensity of activity of EL21 was lower than EL02. In terms of transition probability, 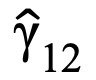 was 0.0082 for EL21 and 0.0097 for EL02, which might suggest that for participant EL21, it was slightly harder to fall asleep when awake than for EL02.

Since the models were fitted individually, we report the estimated parameters for 14 individuals in Table 3. For the wake state, mean 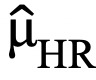 was 87.18 (SD 12.52), while mean 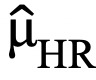 for the sleep state was 66.37 (SD 7.82). The difference in variance in the estimated means in two states among individuals potentially reflected the person-to-person diversity in lifestyle and heart rhythm. Moreover, the individual-specific parameters helped characterize the sleep/wake cycle.

**Table 2:** Estimated parameters in model M2 for EL02 and EL21.

Participant and emission parameters			Wake state	Sleep state
**EL02**				
	**Activity**			
		*µ*_ACT_	2.98	0.27
		*σ* ^2^_ACT_	0.83	0.15
	**Heart rate**			
		*µ*_HR_	110.21	74.39
		*σ* ^2^_HR_	213.68	157.37
	Correlation, *ρ*		0.41	0.04
**EL21**				
	**Activity**			
		*µ*_ACT_	2.22	<0.01
		*σ* ^2^_ACT_	1.08	<0.01
	**Heart rate**			
		*µ*_HR_	80.17	56.46
		*σ* ^2^_HR_	276.27	58.56
	Correlation, *ρ*		0.56	0.01

**Table 3:** Mean estimated HMM parameters for the sample of participants.

Parameters			Wake state, mean (SD)	Sleep state, mean (SD)
**Emission**				
	**Activity**			
		*µ*_ACT_	2.24 (0.29)	0.02 (0.07)
		*σ* ^2^_ACT_	1.16 (0.19)	0.01 (0.04)
	**Heart rate**			
		*µ*_HR_	87.18 (12.52)	66.37 (7.82)
		*σ* ^2^_HR_	241.16 (110.75)	47.18 (36.91)
	Correlation, *ρ*		0.54 (0.07)	0.01 (0.01)
**Transition**				
	Wake state		0.989 (0.003)	0.011 (0.003)
	Sleep state		0.017 (0.002)	0.983 (0.002)

### Investigating the Effect of the Fusion of Heart Rates and Activity in the Model

We compared the final scoring results from approaches based on the heart rate HMM, the activity HMM and our fusion approach minute-by-minute for each participant. Table 4 presents duration, heart rate, and activity level in different combinations of possible results for EL02 as an example.

For EL02, 49.30% (42,599/86,400 minutes) of the recordings were scored as wake, and 33.87% (29,264/86,400 minutes) were scored as sleep by all three methods, which indicated the monotonic relationship between heart rate and activity level for most of the time, whether sleep or wake. There were 13.42% (11,593/86,400) that changed states by our fusion approach compared to that indicated using only one data-source type. Our approach rarely (2/86,400, <0.001%) scored one minute as sleep state if either the heart rate HMM or the activity HMM had classified the minute as wake state. The determination of sleep state in our approach was a combination of activity and heart rate for EL02.

**Table 4:** Comparison between the activity HMM, the heart rate HMM, and our fusion approach for participant EL02.

HMM			Comparison		
Activity only	Heart rate only	Fusion	Duration (total minutes=86,400), n (%)	Heart rate, mean (SD)	Activity level, mean (SD)
wake	wake	wake	42,599 (49.30)	113.37 (11.28)	3.13 (0.91)
wake	wake	sleep	0 (0)	—	—
wake	sleep	wake	2825 (3.27)	76.12 (8.94)	2.14 (0.96)
wake	sleep	sleep	1 (0.00)	74 (—)	0.76 (—)
sleep	wake	wake	8768 (10.15)	101.21 (13.74)	0.82 (0.67)
sleep	wake	sleep	1 (0.00)	96 (—)	0.38 (—)
sleep	sleep	wake	2942 (3.41)	80.72 (8.37)	0.97 (0.68)
sleep	sleep	sleep	29,264 (33.87)	69.62 (5.10)	0.28 (4.24)

For recordings that the activity HMM scored as wake, the heart rate HMM scored as sleep, and our approach scored as wake, the mean heart rate was 76.12, and the mean activity level was 2.14 (equivalent to 7.5 steps per minute). The nontrivial activity level led our approach to score that minute as wake. For recordings that the activity HMM scored sleep, the heart rate HMM scored wake, and our approach scored as wake, the mean heart rate was 101.21, and the mean activity level was 0.82.

**Figure 6:**
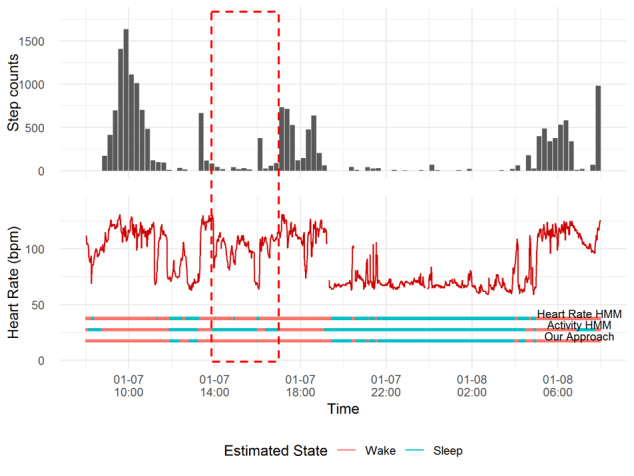
An example plot of observations and scoring results from Heart Rate HMM, Activity HMM, and our approach for EL02. HMM: hidden Markov model.

Furthermore, we present an example of 24 hours of scoring results from the three model types along with observational data for EL02 in Figure 6. The bars below the observations indicated the scored sleep or wake states in three models. From the highlighted period in Figure 6, we can see that if we used activity data alone, it was very likely to be classified as sleep due to the extremely low activity level. However, the high and fluctuating heart rate might suggest the person was awake.

**Figure 7:**
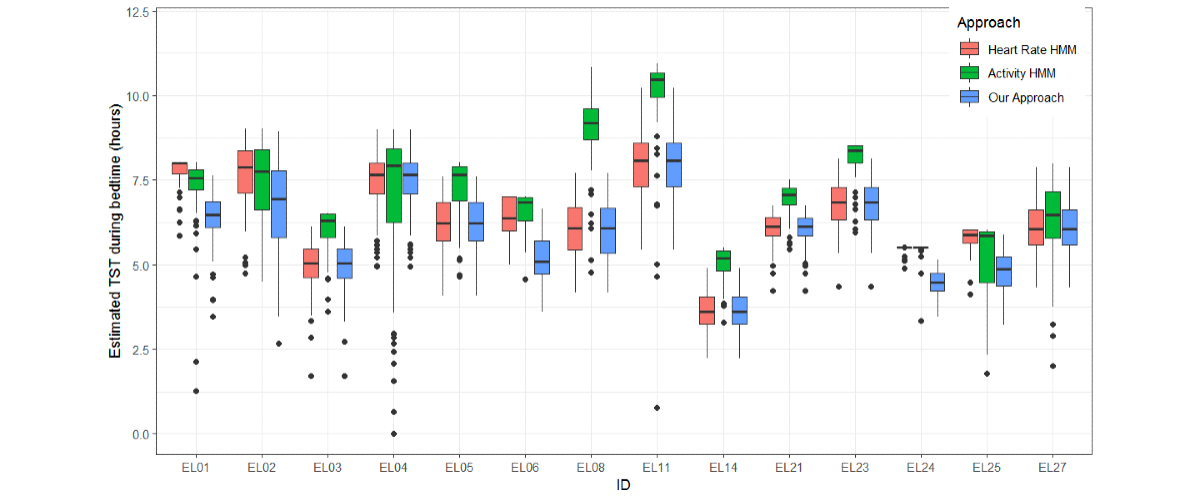
Boxplot of estimated daily total sleep time during bedtime at night from heart rate HMM, activity HMM, and fusion HMM (our approach) for all participants. HMM: hidden Markov model; TST: total sleep time.

Except for epoch-by-epoch comparison, we also evaluated the performance on a crucial sleep metric, the total amount of sleeping time in the recording period [42]. For all participants, we calculated daily total sleep time at night for all available days. The nighttime sleep period of each participant was collected with the Pittsburgh Sleep Quality Index indicating when they usually went to bed at night and got up in the morning [43]. Figure 7 displays the boxplot of estimated total sleep time during their bedtime for all participants. We could see that the median estimated total sleep time at bedtime varied a lot from person-to-person. For EL01, EL02, EL06, EL24, and EL25, the median total sleep time estimated by our approach was less than those from the heart rate HMM and the activity HMM. For the other participant, they were nearly the same as the activity HMM.

### Comparison With Fitbit’s Sleep-Wake Scoring Results

**Figure 8:**
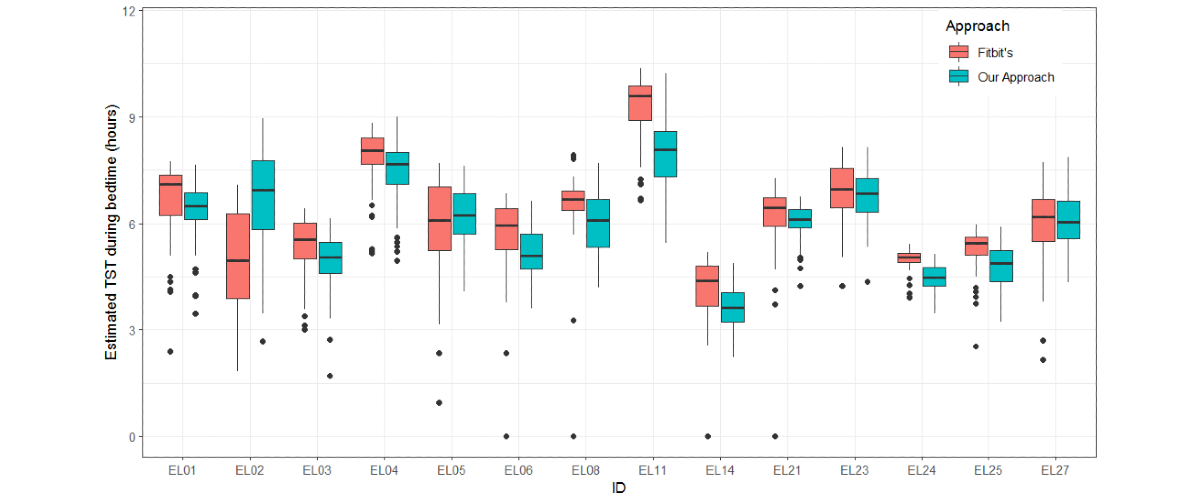
Boxplot of estimated daily total sleep time during bedtime at night from our approach and Fitbit’s approach for all participants. HMM: hidden Markov model; TST: total sleep time.

We compared results from our approach with those from Fitbit’s scoring algorithm minute-by-minute. We treated Fitbit’s sleep/wake scoring algorithm as representative of existing methods. The mean agreement between our approach and Fitbit’s scoring was 87.31%, (range 82.90% to 91.04%). As for total sleep time, the boxplot of the estimated total sleep time at night using our approach and Fitbit’s algorithm are displayed in Figure 8. There were several days where Fitbit’s had no sleep/wake records but had continuous regular heart rate recordings. The median estimates of total sleep time from our approach were lower than those from Fitbit in 12 of 14 participants, which indicated our approach tended to score more wake epochs. The dispersion of estimated sleep duration using our approach was relatively narrower than that of Fitbit’s algorithm, which may suggest a stable indicator of participants’ habitual nighttime sleep duration.

### Investigating Pattern Changes Using Daily Basis Model

The *P* values for independent two-tailed *t* tests comparing estimated parameter values between weekdays and weekends for each person are shown in Table 5. There was no significant difference for γ_21_, *σ*^2^_HR_ for wake state, and *µ_ACT_* for sleep state between weekends and weekdays for any participant. As shown in Table 5, 5 of 14 participants had no significant difference in any of the estimated parameters between weekday models and weekend models, while 9 participants had differences in at least one parameter. For example, EL04 had a significantly lower transition probability from wake to sleep (0.015 for weekdays; 0.018 for weekends). EL11 had a higher mean estimated variance of activity level in wake state on weekdays (1.49 for weekdays; 1.29 for weekends).

**Table 5:** *P* values for *t* tests on estimated parameter values for weekday and weekend for each participant.

ID	*P* values																	
	Transition^a^		Wake state								Sleep state							
			Activity		Heart rate			Corr^b^		Activity				Heart rate			Corr	
	γ_12_	γ_21_	*µ*_ACT_	*σ* ^2^_ACT_		*µ*_HR_	*σ* ^2^_HR_		*ρ*			*µ*_ACT_	*σ* ^2^_ACT_		*µ*_HR_	*σ* ^2^_HR_		*ρ*
EL01	.28	.89	.30	.66		.22	.72		.04^c^			.94	.33		.34	.26		.83
EL02	.64	.94	.90	.85		.44	.99		.74			.90	.54		.63	.55		.86
EL03	.38	.55	.14	.96		.18	.25		.17			.16	.09		.10	.08		.02^c^
EL04	.047^c^	.70	.53	.31		.51	.63		.68			.78	.50		.82	.53		.08
EL05	.45	.58	.64	.48		.91	.39		.69			.66	.74		.70	.03^c^		.68
EL06	.20	.95	.59	.05		.96	.14		.92			.33	.59		.92	.39		.09
EL08	.39	.46	.12	.23		.11	.73		.93			.96	.06		.12	.61		.53
EL11	.70	.70	.63	.002^c^		.60	.16		.17			.18	.42		.73	.43		.63
EL14	.58	.11	.84	.04^c^		.34	.50		.90			.56	.36		.58	.78		.75
EL21	.008^c^	.07	.86	.79		.006^c^	.94		.67			.14	.07		.008^c^	.04^c^		.006^c^
EL23	.97	.85	.52	.56		.56	.62		.54			.28	.43		.32	.62		.63
EL24	.97	.98	.08	.55		.002^c^	.38		.54			.07	.048^c^		.06	.33		.02^c^
EL25	.19	.76	.55	.47		.10	.21		.54			.28	.50		.69	.89		.38
EL27	.66	.54	.009^c^	.08		.30	.44		.01^c^			>.999	.54		.73	.046^c^		.32

^a^γ_11_ and γ_22_ are not reported because they have the same results as γ_21_ and γ_21_.

^b^Corr denotes correlation.

^c^Value is significant *P*<.05.

## Discussion

### Principal Findings

Longitudinal monitoring of sleep duration can objectively help detect sleep disorders and reduce the risk of related diseases. In order to facilitate personalized home-based monitoring, it is essential to record sleep and wake states using wearable devices efficiently and nonintrusively. In this study, we proposed a novel personalized and unsupervised approach for sleep/wake identification using both heart rate and activity data from a commercial wearable device. The approach was successfully implemented in case studies of community-dwelling older adults.

Our proposed approach is the first unsupervised and personalized sleep/wake classification approach, to our knowledge. It does not require any time-consuming and costly PSG annotation, which is hard to obtain simultaneously with wearable device data [15,36]. Furthermore, our approach was efficient enough to be adaptive to a different participant without requiring PSG annotations. In our case study, the variance in estimated parameters in HMM between participants also proved the necessity of a personalized model.

The data-level fusion of activity and heart rate data for sleep/wake scoring in wearable device was explored. Based on comparison among scoring results from HMMs using heart rate only, activity data only, and both data sources, we concluded that our approach could potentially help identify more wake epochs for people who have distinguishable heart rate patterns between sleep and wake. This coincided with the findings of significant different heart rate during sleep and wake states [32] and its classification power in sleep/wake identification algorithms [33,44].

Our approach had results that were mostly consistent with those of Fitbit, a commonly used commercial device. For most of the participants, our approach tended to score more wake epochs during bedtime, which may be potentially useful as it has been shown by Montgomery-Downs et al [45] that many commercial wearable devices, when compared to PSG, tend to overestimate sleep epochs. This should be further investigated with PSG annotations.

### Strengths

In addition to sleep and wake identification and total sleep time estimation results, this paper proposed an approach that provides a new probabilistic way to characterize and quantify activity patterns and cardiac patterns during sleep and wake for each participant with estimated HMM parameters. A low estimated mean activity in wake states suggest a sedentary behavior style when the participant is wake, which should be of concern to the participant or their health care provider [46]. Abnormally high estimated mean heart rate in sleep states can be an indicator of autonomic nervous system dysfunction or of the development of chronic fatigue syndrome [47]. A high estimated probability of transitioning from sleep to wake might suggest disturbed sleep. Monitoring these parameters for clinical use is promising and remains to be explored in specific tasks.

In addition, we demonstrated how to characterize cardiac and behavior patterns on a daily basis. During our 3-month study, some participants exhibited significantly different patterns between weekdays and weekends. On weekdays, participants had regular visits to older adult centers in their community, which provided various activities. This could explain why some participants tended to have higher estimated mean and variance of activity level for wake state and were less likely to transition from wake to sleep on weekdays. There exists not only interindividual variability in sleep patterns but also intraindividual variability [34]. This analysis can also provide a reference to improve the accuracy of the personalized model to infer sleep/wake states by including covariates (eg, weekday or weekend) in the transition matrix or emission distributions in HMM.

### Limitations

We acknowledge that there are also some limitations to our work. First, direct conclusions about our approach’s accuracy cannot be drawn because of the lack of PSG recordings in this study and the small sample size. The estimated sleep/wake states might also be better interpreted as resting-active states at this stage. Second, the comparison with Fitbit’s scoring may not be very fair since the exact algorithm is not publicly known and the data type used for sleep/wake scoring was unknown as well. We also cannot reasonably compare the proposed approach with existing methods on the same data set because of the lack of personalized and unsupervised approaches in sleep/wake scoring (to our knowledge). Third, we collected heart rate and step count data using Fitbit, which were reported in different granularities. In order to achieve data-level fusion and prepare it for modeling, we simulated 1-minute step data from 15-minute step data which might have resulted in some imprecision. We strongly call for different reporting granularity options in commercial wearable devices to further facilitate research and their use in health care monitoring systems.

### Future Work

In the future, we plan to compare the proposed approach with PSG to further validate the accuracy of the scoring. Some future research directions include (1) exploring the relationship between the length of data and accuracy of sleep/wake classification to yield a reliable algorithm, (2) exploring the existence of not only interindividual variability in sleep patterns but also intraindividual variability [34] (such as developing an incremental approach for daily sleep/wake duration reporting where the sleep/wake pattern can be re-estimated as new data are captured), (3) with the strength of HMM, it would be interesting to see whether sleep characteristics shown in periodically estimated HMM parameters can be correlated with health condition or circadian rhythm changes, and (4) exploring the association between subjective Pittsburgh Sleep Quality Index and estimated HMM parameters in different populations.

## Abbreviations

AIC: Akaike information criterion
BIC: Bayesian information criterion
HMM: hidden Markov model
PSG: polysomnography
QQ: quantile-quantile
